# Factors Which Influence Owners When Deciding to Use Chemotherapy in Terminally Ill Pets

**DOI:** 10.3390/ani7030018

**Published:** 2017-03-07

**Authors:** Jane Williams, Catherine Phillips, Hollie Marie Byrd

**Affiliations:** 1 Animal Health Research Group, Hartpury University Centre, Gloucester GL19 3BE, UK; 2 Veterinary Nursing Research Group, Hartpury University Centre, Gloucester GL19 3BE, UK; Catherine.phillips@hartpury.ac.uk (C.P.); hollie1111@aol.com (H.M.B.)

**Keywords:** veterinary medicine, oncology, client decision-making, cancer, pets

## Abstract

**Simple Summary:**

Cancer is as common amongst pets as it in humans. Chemotherapy can be integrated into treatment regimes for terminally ill pets to attempt to shrink tumours to extend life expectancy, but it does not cure cancer and it can have negative side effects including vomiting, depression and behavioral changes. To date, little research has been undertaken to explore owners’ decisions whether or not to treat their animals with chemotherapy. Seventy-eight dog and cat owners completed an online questionnaire to determine if they would opt for chemotherapy if their pet was diagnosed with cancer, and asked how they thought their pet’s quality of life would be affected. Fifty-eight percent of respondents would not use chemotherapy largely due to their previous experience of it. Seventy-two percent over estimated pet survival time post chemotherapy, with most people believing it would lead to remission or a cure. Owners expected their pets to be less active, sleep more and play less, reducing their quality of life. Common side effects associated with chemotherapy were not rated as acceptable. The results suggest pet owners would benefit from an increased understanding of the positive and negative impacts of chemotherapy when initially discussing treatment options with the veterinary team.

**Abstract:**

Chemotherapy is a commonly integrated treatment option within human and animal oncology regimes. Limited research has investigated pet owners’ treatment decision-making in animals diagnosed with malignant neoplasia. Dog and cat owners were asked to complete an online questionnaire to elucidate factors which are key to the decision making process. Seventy-eight respondents completed the questionnaire in full. Fifty-eight percent of pet owners would not elect to treat pets with chemotherapy due to the negative impact of the associated side effects. Seventy-two percent of respondents over estimated pet survival time post chemotherapy, indicating a general perception that it would lead to remission or a cure. Vomiting was considered an acceptable side effect but inappetence, weight loss and depression were considered unacceptable. Owners did expect animals’ to be less active, sleep more and play less, but common side effects were not rated as acceptable despite the potential benefits of chemotherapy. Based on the results, veterinary teams involved with oncology consultations should establish if clients have prior experience of cancer treatments and their expectations of survival time. Quality of life assessments should also be implemented during initial oncology consultations and conducted regularly during chemotherapy courses to inform client decision making and to safe guard animal welfare.

## 1. Introduction

Chemotherapy is a commonly integrated treatment option within human and animal oncology regimes [1,2,3] which aims to reduce the growth of tumours and spread of malignant neoplastic cells. It is defined as the ingestion or injection of cytotoxic drugs to destroy neoplastic or cancer cells [4]. In animals five cancer treatment approaches are taken: palliative care, surgery, chemotherapy (±surgery), radiotherapy or euthanasia [5]. Within veterinary practice, the treatment approach for animals diagnosed with malignant and terminal neoplasia will be informed by discussion with the veterinary team including an overview of therapeutic options, their side effects and benefits, prognosis and survival time, and impact on the pet’s quality of life. Despite this, research suggests chemotherapy is often elected for by pet owners due to their prior experience or knowledge of its positive (perceived effective) application in human cancer treatment [6,7].

Neoplasia affects approximately 0.02% dogs and 0.01% cats in the UK per annum [8,9]. The prognosis for animals suffering from malignant neoplasia varies upon the clinical approach taken but generally is poor. For example, the average survival time post-diagnosis for dogs with lymphoma without treatment is 4 to 6 weeks [10]. Research has shown this can be extended with treatment, for example Wang et al. [11] reported survival times of between 5 and 7.5 months with treatment [11]. It should however be noted that survival times will vary between individuals, the type of malignancy present and the oncology regime undertaken.

The decision making process during oncology treatment can lead to ethical dilemmas for owners and the veterinary team. The status of animals in society is inconsistent, ranging from owners who treat their pets as family members [12] and will spare no expense upon them, to others who will abandon animals when they become an inconvenience [13]. The Royal College of Veterinary Surgeon’s Code of Professional Conduct requires veterinary surgeons to provide owners of animals undergoing chemotherapy with an outline of all potential treatment options, associated fees and side effects, and the subsequent prognosis for their pet [14]. The priority for the veterinary team is to safeguard the welfare of their animal patient but they also need to support the owner/s through an emotive decision. However, the owner ultimately has the responsibility to make a decision which safeguards the welfare of their pet [15] and as such needs to understand the full implications of their choice. Within human medicine, terminally ill patients and their care givers often feel doctors have ‘given up’ on them if chemotherapy is not offered as treatment option or is withdrawn during the latter stages of palliative care [16]. A similar scenario may exist within veterinary medicine but this has not been investigated to date. Interestingly, Giuffrida and Kerrigan [17] report that the owners of terminally ill pets value the quality of life of their pet over extended survival times. This suggests that an open and transparent approach from the veterinary team when discussing with the owner the risks, benefits, impact of the treatment involved and the outcome with consideration of quality of life, is essential to support informed consent for subsequent treatment protocols or if they decide to opt for euthanasia [18,19,20].

### Chemotherapy Treatment

The use of chemotherapy within therapeutic oncology programmes in the veterinary sector is becoming more commonplace [21]. Despite this no large scale studies to date have investigated the prevalence, incidence or severity of potential side-effects associated with the treatment in animals, or if clinical symptoms reported as side effects may be due to alternative aetiologies. Therefore owners may refer to human medicine and their own experiences to judge the impact of chemotherapy protocols within their pets. A full review of the impact of chemotherapy on survival times in animals is beyond the scope of this review due to the range of different protocols, multitude of cancer types and confounding factors which can affect prognosis. Illustrations of survival times are provided however readers unfamiliar with oncology are advised that these provide specific examples and do not represent the entirety of the field. Chemotherapy has been shown to extend the lifespan of terminally ill dogs diagnosed with malignant neoplasia on average by 185 days (histiocytic sarcoma) [22], 301 days (appendicular osteosarcoma) [23] and 216 to 342 days (lymphoma) [24]. Similar results have been found in cats that have undergone chemotherapy for extranodal lymphoma recording survival times ranging from 70 days to 749 days [25]. Surgical removal of malignant neoplastic masses without an accompanying chemotherapy course can also exert a beneficial impact on survival, however survival time is often reduced compared to animals with the same diagnosis who undergo surgery and chemotherapy (mammary carcinoma) [26]. Therefore, chemotherapy is considered to extend survival time for the terminally ill pet compared to surgical reduction alone and has a clear benefit as part of an oncology treatment regime [27]. Weeks et al. [28] surveyed the expectations of terminally ill patients with lung and colorectal cancer, finding 69% and 81% of patients respectively felt that chemotherapy would cure their cancer. Prior experience of chemotherapy may influence pet owners’ decision making and although surveys suggest quality of life is of key importance [17], when faced with losing their pet, extended survival times, which chemotherapy offers, alongside the incorrect perception that chemotherapy could facilitate a cure, may influence owner choices.

Chemotherapy can cause detrimental side effects in animals [29] and humans [28]. The treatment is non-discriminatory and can have a toxic effect on diseased and healthy tissue [30], resulting in myelosuppression, gastrointestinal toxicosis and nausea in human patients [31,32,33]. Within dogs, analogous side effects have been reported post chemotherapy treatment including gastrointestinal disease, myelosuppression, cardiotoxicity, dyspnoea and neutropenia resulting in immunological challenges predisposing patients to secondary infections [34,35]. Cognitive changes are also often reported by human patients post chemotherapy treatment, termed “chemobrain”; symptoms include difficulty comprehending normal tasks, lack of concentration, memory issues and lack of awareness of what they are doing [36]. Canine patients will often vomit after treatment and are thought to experience nausea leading to anorexia during chemotherapy [37,38]. As a result of regular emesis, patients can experience weakness, fatigue, anaemia and fever-type symptoms which can result in weight loss. Behavioural changes, reported within quality of life assessments in neoplasia cases [29], with pets becoming progressively more lethargic and depressed (unresponsive, inactive and withdrawn with altered eating and sleeping patterns) resulting in reduced interaction with their owners and the environment which can be upsetting for owners. However it should be remembered that the remit of chemotherapy is to shrink or prevent spread of tumours, and many of the perceived negative side effects should be considered alongside the beneficial effect this treatment can have on prognosis and survival time in canine and feline cancer patients.

Quality of life assessments are often employed to make judgments on an animal’s welfare status [29,39,40]. Yeates and Main [41] advocate that a quality of life assessment should be included by the veterinary team when considering therapeutic options for animals suffering from malignant neoplasia. Integrating Quality of Life (QOL) assessments can inform owner decision making, to ensure an ethical balance between *quality* and *quantity* of life is achieved, and the welfare of the animal affected is fully considered [40,41]. The quality of life process can also provide a baseline measure for subsequent assessments. Quality of life assessments, conducted via owner questionnaires, have been conducted in cats [39,42] and dogs undertaking chemotherapy [43,44]. Generally, owners across species were very perceptive to clinical changes in their pets but did not appear to demonstrate equal acuity when identifying QOL changes. This may reflect the tool used, as many quality of life assessments rarely progress beyond evaluation of clinical parameters and fail to integrate other qualities such as cognition, normal functionality, and con-specific interaction within them [44]. 

To date, research evaluating chemotherapy use in pets has centred on retrospective reviews of veterinary records to evaluate clinical symptoms, therapeutic approaches and subsequent survival times in neoplastic animals. For examples refer to Finlay et al. [45] and Wright et al. [19]. Whilst this information is critical to enable informed dialogue from the veterinary team to the client, assessment of the factors which influence the decision making process pet owners undertake when deciding whether to consent to a course of chemotherapy has been neglected. Therefore, this study aimed to explore factors which may influence owners’ decision to elect to undertake chemotherapy in animals, to help inform the approaches taken by the veterinary profession.

## 2. Method

A mixed methods approach was used to survey current and previous owners of dogs or cats in the UK, to ascertain what factors would influence the decision making process to elect to undertake chemotherapy treatment in a terminally ill pet. Ethical approval was obtained from the University of the West of England (Hartpury) Ethics Committee (Project Identification Code: ETHICS2016-04).

### 2.1. Participants

Participants were recruited via social media: Facebook and Twitter, between November 2015 and February 2016 due to the capacity for social media to acquire a large number of participants [46]. Subjects were required to be over 18 years of age and to currently own or have previously owned a cat or dog, to be eligible for participation. No previous experience with neoplasm in terminally ill animals or humans was essential. Only fully completed questionnaires progressed to data analysis.

### 2.2. Survey Design

A questionnaire was designed in Google Forms™. The questionnaire included open and closed questions (Supplementary Materials). Likert scales were also integrated within the questionnaire to allow participants to rank the importance of key themes surveyed. These were complemented by the use of open questions to encourage participants to answer with unprompted responses to facilitate a truer expression of the emotions and feelings they had on the use of chemotherapy in animals [47]. Three key themes were adapted from previous research [39,40,43] and were embedded within the questionnaire: owner perception of the benefits and side effects of chemotherapy (Table 1), rating of quality of life (scale 1: low to 10: high) pre, peri and post chemotherapy, and views on survival times and life expectancy with and without chemotherapy treatment.

### 2.3. Data Analysis

Data from completed questionnaires were transferred to Microsoft™ Excel version 2013 (Microsoft Corporation, Redmond, WA, USA). Frequency analysis of categorical data was performed across the cohort, then data were organised by gender and age within gender to highlight any emergent trends within the subgroups. Grounded theory analysis was applied to the narrative obtained from open questions to enable emergent themes from the data to be recognised [48].

## 3. Results

**Figure 1:**
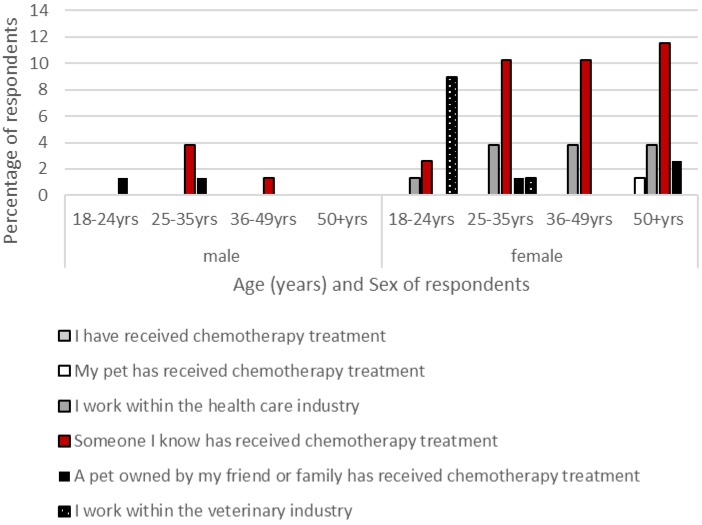
Participants’ previous experiences with chemotherapy treatment (CT).

A total of 78 questionnaires were completed in their entirety and went forward to analysis. The majority of respondents were female (*n* = 68); these represented a variety of age ranges: 18–24 years: 23%, 25–35 years: 26%, 36–49 years: 27% and over 50 years: 22%. Male participants (*n* = 10) demonstrated an older age demographic: 18–24 years: 10%, 25–35 years: 40% and over 50 years: 50%. The majority of respondents had some prior experience of chemotherapy (female respondents: 53%; male respondents: 60%), this was within a friend or family member for 39% of female respondents and 40% of male respondents, and in a pet for 14% of female respondents and 20% of male respondents (Figure 1). Fifty-five percent of respondents (female respondents: 57%; male respondents: 30%) stated they were familiar with the side effects that accompanied chemotherapy in animals, with slightly more, 57%, aware of the side effects associated with chemotherapy in human patients (female respondents: 61%; male respondents: 40%).

**Figure 2:**
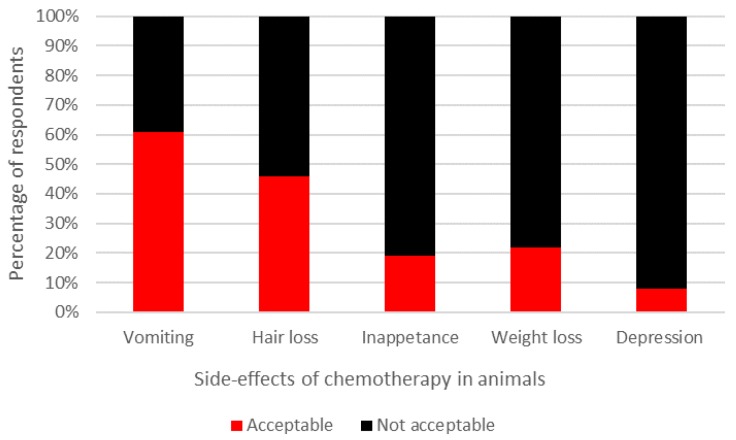
Acceptability of side effects associated with chemotherapy treatment in dogs and cats.

**Figure 3:**
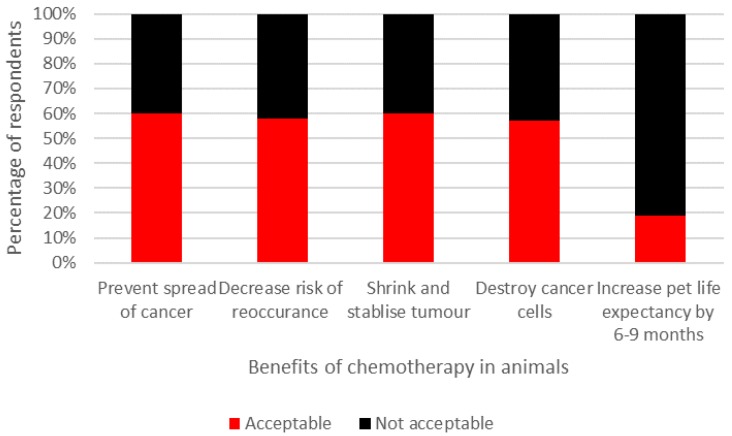
Acceptability of beneficial effects associated with chemotherapy treatment in dogs and cats.

**Figure 4:**
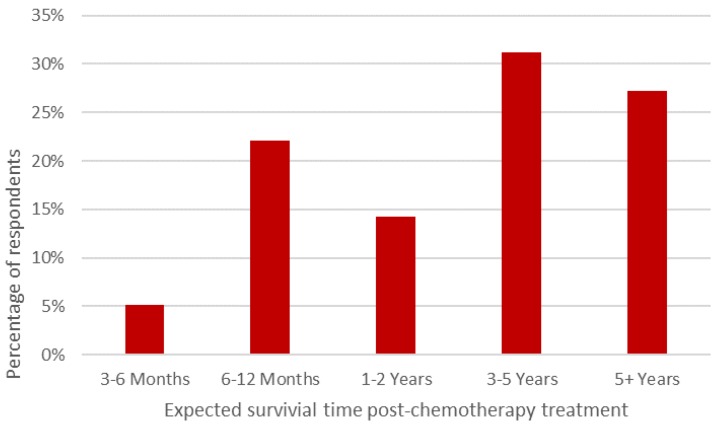
Perceived survival time post-chemotherapy treatment in dogs and cats.

The majority of participants (58%) believed that the benefits of chemotherapy did not counterbalance the impact of the potentially negative side effects which animals may experience during treatment. However, when respondents were asked to rate the acceptability of defined side effects and benefits of chemotherapy, mixed opinions were recorded (Figure 2 and Figure 3, respectively). Interestingly, respondents thought that pets’ QOL would be enhanced after chemotherapy (median rating: 7; interquartile range (IQR): 2) compared to prior to (median rating: 5; IQR: 3) and during treatment (median rating: 5; IQR: 3). All respondents indicated that they believed chemotherapy would extend an animal’s life expectancy and 72% felt that chemotherapy would extend survival time over one year (Figure 4).

**Figure 5:**
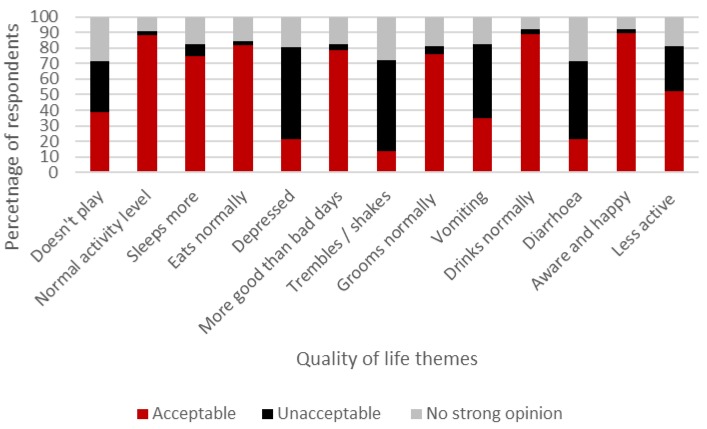
Dog and cat owners’ expectations of pets’ quality of life during and after chemotherapy.

Respondents expectations on the quality of life dogs and cats experience during chemotherapy regimens varied (Figure 5). The results indicate the majority of owners wanted their animal to retain a *normal* quality of life with regards to eating, drinking, behaviour and activity levels, and low expression of known side effects associated with chemotherapy: vomiting, diarrhoea and depression.

When asked if survival time would influence the decision to undertake chemotherapy in their pet, a total of 52 out of 78 respondents (67%) disagreed, or strongly disagreed to the statement “I would opt for chemotherapy treatment if my pet will live for an extra 3 months with the chemotherapy”. Generating mixed comments such as “*cancer if the lesser evil*” and “*months is enough time as long as it is enjoyable*”. Whilst 40 respondents (52%) agreed, or strongly agreed to the statement “*I would opt for chemotherapy treatment if my pet will live for an extra 12 months with chemotherap*y”, although others felt: “*one year is not enough*”. Forty-two percent of participants (45% female; 30% male) would elect for chemotherapy if their pet was diagnosed with a malignant tumour knowing the potential benefits of the treatment. This reduced to 35% (35% female; 40% male) when considering the side effects of chemotherapy treatment. These participants generally believed the “*benefits (of chemotherapy) outweigh the side effects*” “*it (chemotherapy) will give the animal vital time*” and “*the side effects don’t appear to be drastic*”, “*any cancer treatment should be available to animals*”, “*chemotherapy is a wonderful idea even with limited knowledge*”, with one commenting “*anything is better than putting the animal to sleep*”. In contrast, respondents who would not elect for chemotherapy in their pet felt “*that after seeing what it does to a person, I am unsure as to how ethical it is to do this to an animal that doesn’t understand*” and, “*having seen a relative undergo chemo, I would be less inclined to agree to chemo for my dogs*”. When making a decision to treat animals with CT, the use of prior knowledge obtained by the participants exerted an influential impact on some respondents’ decision making: “*after seeing what it does to humans, I don’t think it is ethical*”, “*I would find it hard as my sister and niece have been through it*”, “*cancer is the lesser evil*” and “*surgery is a quicker resolution*”*.*

## 4. Discussion

Most of the participants had some experience of chemotherapy in humans and/or animals, which appears to inform decision making when considering if they would elect to place a terminally ill pet upon a course of chemotherapy. Previous experience has been identified to contribute to the decision making process in human medicine [49,50,51]. The results here suggest that owners’ beliefs and prior experience of chemotherapy, especially their perception of side effects over potential benefits of the treatment, will influence their decision to use chemotherapy in their pets. It would be worthwhile for veterinary teams to allocate time to fully understand owners’ historic experiences of chemotherapy as these will influence the treatment regime selected.

Similar traits to those observed in human oncology patients and their carers appear to also occur within animal owners. The majority of respondents believed their pet’s quality of life would improve post-chemotherapy but many (72%) overestimated average survival time post-treatment. Fewer owners would have elected for chemotherapy if the result was a shorter 3-month extended survival compared to a 12-month survival period for their pet, which suggests quality of life is a key consideration in decision-making. However it should be noted that “agree” and “strongly agree” responses were summed to obtain these figures, therefore the results may over represent the strength of feeling of those who took part. Respondents also quantified the benefits of chemotherapy in terms of the treatment being a cure rather a palliative intervention. These opinions mimic those found in human cancer patients and their caregivers [28]. Human cancer patients have indicated a preference to be fully informed before undertaking treatment to provide time to adapt to their diagnosis and make a fully informed decision [52,53,54]. A similar approach would be advocated in pet owners as proxy representatives of the animal, as our results suggest that owners consider what treatment approach to adopt from their perspective rather than their pets. It is therefore also important to eliminate any potential disconnect that exists between owners’ perception of the severity of side effects and the reality of what to expect within chemotherapy regimens. Veterinary teams should ensure their approach in terminal cases includes knowledge transfer of the fundamental characteristics and effects of chemotherapy, with a clear focus on how an animal’s quality of life will be affected and the likely incidence and expression of side effects, to enable owners to possess sufficient knowledge and understanding to make a fully informed decision. Paradoxically in human chemotherapy, patients who feel they have a good relationship with open communication with their clinician are at higher risk of having unrealistic expectations of the long term benefits of chemotherapy [28]. Therefore, it would be beneficial to integrate a period of reflection into the decision making process and perhaps a multi-person approach is warranted to ensure clients engage with information transfer. Without these opportunities, owners may select chemotherapy to facilitate more time with their pet, despite a poor understanding of the impact of chemotherapy on the animal which could lead to increased distress during treatment protocols and after the animal’s death akin to feelings observed in human care givers post-bereavement [16].

The majority of respondents expected aspects of an animal’s quality of life to reduce during and after a course of chemotherapy treatment, with pets sleeping more, being less playful and showing reduced activity levels. Interestingly, despite 55% of respondents stating they were aware of the side effects associated with chemotherapy in animals, most owners did not feel these side effects were acceptable when judging a pet’s quality of life. Respondents also did not feel the common side effects of chemotherapy (vomiting, diarrhoea and behavioural changes) would be acceptable, as well as wanting affected animals to experience more good than bad days. Previous work [46] found that although owners of animals undergoing chemotherapy were very perceptive of clinical changes they did not recognise signs which represented a reduced quality of life, despite the same people prioritising quality of life over extended life expectancies [17]. The results suggest a disconnect exists between owners’ expectations of what their pet’s quality of life should be during chemotherapy and what it is likely to be. Similarly, most owners here equated the therapy with an extended survival time beyond average life expectancies post diagnosis or that it represented a cure, commenting: “(chemotherapy) *saves them, extends life, is a cure, chemotherapy increases their quality of life, the benefits outweigh the side effects, and having side effects for a short time and to live a lot longer* and *healthier is better*”. A similar paradox occurs within human oncology [16,28] with misconceptions of the clinical impact of chemotherapy (quality of life and life expectancy) common amongst patients receiving palliative care and their care-givers. It is imperative to note that chemotherapy can lengthen survival times and offer a better quality of life for neoplastic animals, a concept that should not be lost in client-veterinary communication. Therefore it is important that the veterinary team supporting decision making in owners and understand that such paradoxes exist as they could potentially misinform a client’s judgement of a pet’s quality of life and welfare, and the subsequent decision to not elect for chemotherapy or to agree to the treatment.

Quality of life assessments are key tools that can be used to benefit patients, clinicians, caregivers or owners, and can inform the medical/veterinary therapeutic decision making process [55]. The majority of assessments are disease specific and tend to focus on clinical parameters [39]. Examples in human medicine include the EuroQol [56], Sickness Impact Assessment (SIP) [57] and the SF-36 [58]. Readers are recommended to refer to Vols et al. [29] and Belshaw et al. [40] for reviews of quality of life tools used within veterinary medicine. Specific oncology quality of life assessment tools are also available (for example: De Haes et al. [59]). There is general agreement that an effective quality of life assessment should contain six fundamental dimensions [59]:

1. Physical functioning (physiological, biomechanical and neural parameters, and the ability to perform routine tasks—quantitative assessment),
2. Psychological functioning (cognitive abilities, mental health status, mood and personality—quantitative and qualitative assessment). It should be noted these facets are difficult to assess within animals and general behavioral changes may need to be considered to provide a measure of psychological function,
3. Social functioning (environmental interaction and relationships—qualitative assessment),
4. Role activities (motivation, communication, play and exercise—quantitative and qualitative assessment),
5. Overall life satisfaction (enjoyment and fulfilment—qualitative assessment), and,
6. Perception of health status (overall rating—quantitative and qualitative assessment).

The key goal of quality of life assessment as a tool to inform owners’ decision making prior to electing for an animal to start chemotherapy treatment or during chemotherapy regimens, is the ability of the assessment to quantify clinically meaningful changes. Clinically meaningful changes can be defined as those which influence a patient’s management or that result in a reduction or improvement in functionality, or an increase or decrease in clinical symptoms [55]. Interpretation of change can in itself be challenging as it is difficult to quantify if a change in score of ±0.1 or ±1.0 equates to a clinically meaningful change. In veterinary oncology, quality of life assessments are predominately questionnaire based and rarely go beyond clinical parameters [47]. More work needs to be done to define reliable and valid tools and parameters which capture the full repertoire of changes that occur in an animal’s quality of life, specifically here with reference to the terminally ill oncology patient [17]. The development of effective quality of life assessments would provide a baseline measure to facilitate comparison between individuals in the clinical environment. Additionally, quality of life assessments should be used as a precursory tool to facilitate client-veterinary discussions over the prognosis and treatment options available, and enable clients to make truly informed decisions whether selecting palliative care or euthanasia [17]. Therefore, we would recommend that veterinary teams integrate a quality of life assessment into the initial consultation process for the oncology patient and implement regular quality of life assessments for the duration of their treatment to optimise animal welfare.

The study does have limitations as questionnaires cannot fully explore the complexity that underpins the perception and feelings of the respondents surveyed. The results presented here represent the views of this sample and their perception of chemotherapy as a treatment, and these may not be an accurate representation of broader pet owners’ feelings and opinions. Further work incorporating larger numbers would be warranted. Future research using focus groups, interviews and case studies drawn from individuals who were or had experienced chemotherapy directly with their pet is essential to gain a broader insight into the owner decision making process, owner views of chemotherapy and its impact on pets, and the influences of the veterinary team upon this.

## 5. Conclusions

The veterinary team has a duty of care to their clients and their pets to ensure the full complexities of oncology and chemotherapy are communicated during consultations. Prior experience of chemotherapy appears to directly influence owner decision making when considering whether to undertake a course of chemotherapy in a pet. Owners generally overestimated the impact of chemotherapy on pet survival times post treatment potentially establishing false expectations which could result in enhanced distress post bereavement. Owners rate pets’ quality of life as key when choosing whether to engage with chemotherapy, however this assessment appears focused on clinical parameters and not functional tasks, personality expression or changes in behaviour. Based on the results, veterinary teams involved with oncology consultations should establish if clients have prior experience of cancer treatments and their expectations of survival time. Quality of life assessments which evaluate patient health status via clinical parameters, physical, psychological and social function, interaction and life satisfaction should also be implemented during initial oncology consultations and conducted regularly during chemotherapy courses to inform client decision making and to safe guard animal welfare.

## Figures and Tables

**Table 1:** Quality of life assessment questions.

Participants Were Asked to Rate if They Found the Potential Impact of Chemotherapy in Animals, for the Questions Listed, as Acceptable or Unacceptable.
My pet does not play during chemotherapy
My pet’s activity is the same during chemotherapy
My pet sleeps more than usual during chemotherapy
My pet eats normally during chemotherapy
My pets seem depressed during chemotherapy
My pet has more good days then bad during chemotherapy
My pet trembles and shakes occasionally during chemotherapy
My pet grooms normally during chemotherapy
My pet experiences vomiting during chemotherapy
My pet drinks normal amounts during chemotherapy
My pet has diarrhoea during chemotherapy
My pet is aware and happy when I’m present during chemotherapy
My pet is less active during chemotherapy

## Supplementary Materials

The following are available online at www.mdpi.com/2076-2615/7/3/18/s1, Questionnaire Design.
