# Supplementary material for: Factors Which Influence Owners When Deciding to Use Chemotherapy in Terminally Ill Pets
Source: Animals (Basel). 2017 Mar 7;7(3):18. doi: 10.3390/ani7030018 (PMC5366837; doi:10.3390/ani7030018)
Supplement: Supplementary file 1 [file animals-07-00018-s001.pdf]

Supplementary Materials: Factors Which Influence Owners When Deciding to Use Chemotherapy in Terminally Ill Pets

Jane Williams, Catherine Phillips and Hollie Marie Byrd

Questionnaire Design

The following document presents the questions asked to respondents and provides an outline of the formatting, however it should be noted the questionnaire was undertaken online via Survey Monkey, therefore some formatting differences do exist.

Introduction

We would like to invite you to take part in a research study looking at how owners perceive the use of chemotherapy in cats and dogs which have been diagnosed with terminal cancer. Chemotherapy treatment is widely used in human medicine as part of the treatment approach in cancer patients. It is also becoming more popular as a treatment option in the veterinary industry. Understanding how owners feel about the use of chemotherapy in terminally ill dogs and cats, can help inform how veterinary practices approach initial discussions with clients at what is often an emotionally charged time.

The questionnaire should not take more than 10 minutes to complete. Please note, there are no right or wrong answers, we are interested in your opinions and feelings.

Please note that all information which is collected during the course of the research will be kept on a password protected laptop with the research. All information will anonymous. Questionnaire data will be held but it will all be coded for anonymity.

Section 1: Personal Information

1. Are you male or female? Please tick the most appropriate box:

**Male**	**Female**

2. What is your age? Please tick the most appropriate box:

**18–24 years**	**25–35 years**	**36–49 years**	**50+ years**

3. Do you own a cat or dog? Please tick the most appropriate box:

**Cat**	**Dog**

4. Have you ever had a pet which has been diagnosed with cancer, and did they receive chemotherapy? Please tick the most appropriate box:

**Yes**	**No**	**Yes**	**No**
**Cancer diagnosis**		**Chemotherapy treatment**	

5. Have you had any personal experience of chemotherapy? Please tick the most appropriate box:

**Yes**	**No**

If you answered Yes, please tick all boxes which apply to you from the list below:

	**I have received chemotherapy treatment**
	**Someone I know has received chemotherapy treatment**
	**My pet has received chemotherapy treatment**
	**A pet owned by my friend/s or my family has received chemotherapy treatment**
	**I work within the health care industry**
	**I work within the veterinary industry**
	**Other, please specify.**

Section 2: Chemotherapy Treatment

1. Please select (tick) which answer most closely describes how you feel about the statement:

**Tick the box which most closely describes how you feel about the following statements**	**Yes, very familiar, extensive knowledge/experience**	**Familiar, some knowledge /experience**	**Unfamiliar, not sure if knowledge/ experience is correct**	**Very unfamiliar, no experience or knowledge**
Are you aware that chemotherapy can be used as part of the treatment protocol for animals diagnosed with cancer?				
Do you know what the benefits of chemotherapy are for humans?				
Do you know what the side effects of chemotherapy are for humans?				

2. In your own words, how would you describe how you feel about the use of chemotherapy treatment in cats and dogs diagnosed with terminal cancer?

3. Benefits of chemotherapy, please select (tick) which answer most closely describes how you feel about the statement:

**Tick the box which most closely describes how you feel about the following statements**	**Very acceptable**	**Acceptable**	**Neither acceptable or unacceptable**	**Unacceptable**	**Very unacceptable**
**Chemotherapy prevents the spread of cancer**					
**Chemotherapy decreases the risk of cancer reoccurring**					
**Chemotherapy shrinks and stabilises (prevents further growth) the tumour**					

**Chemotherapy kills cancer cells alongside other treatments**					
**Chemotherapy can extend life expectancy by 6-9 months (post cancer diagnosis)**					

4. Side effects of chemotherapy, please select (tick) which answer most closely describes how you feel about the statement:

**Tick the box which most closely describes how you feel about the following statements**	**Very acceptable**	**Acceptable**	**Neither acceptable or unacceptable**	**Unacceptable**	**Very unacceptable**
**Chemotherapy can cause vomiting**					
**Chemotherapy can cause hair loss**					
**Chemotherapy can cause inappetence (lack of appetite)**					
**Chemotherapy can cause weight loss**					
**Chemotherapy can cause weakness and lethargy**					
**Chemotherapy can cause depression**					

5. In your opinion, would you say the benefits associated with chemotherapy treatment counter balance the common side effects associated with chemotherapy?

**Yes**	**No**

If you ticked yes, please state why you believe the benefits of chemotherapy counter balance the side effects of chemotherapy:

If you ticked no, please state why you believe the benefits of chemotherapy do not counter balance the side effects of chemotherapy:

6. Your pet has been diagnosed with terminal cancer and you have elected to engage with a course of chemotherapy treatment. Below is a list of statements that describe your pet's quality of life during chemotherapy, please select (tick) which answer most closely describes how you feel about the statement:

**Tick the box which most closely describes how you feel about the following statements**	**Very Acceptable**	**Acceptable**	**Neither acceptable or unacceptable**	**Unacceptable**	**Very Unacceptable**
My pet does not play as much as normal during chemotherapy					

My pets activity is the same as normal during chemotherapy					
My pet sleeps more than usual during chemotherapy					
My pet eats normally during chemotherapy					
My pets seem depressed during chemotherapy compared to normal					
My pet has more good days then bad days during chemotherapy					
My pet trembles and shakes occasionally during chemotherapy					
My pet grooms normally during chemotherapy					
My pet experiences vomiting during chemotherapy					
My pet drinks normal amounts during chemotherapy					
My pet has diarrhoea during chemotherapy					
My pet is aware and happy when I'm present during chemotherapy					
My pet is less active during chemotherapy					

7. Quality of life describes how multiple factors (for example, exercise level, happiness, interaction) come together to contribute to the general well-being of your pet.

Please rate your expectation of your pet's minimum quality of life (1 = poor quality of life and 10 = excellent quality of life) for the statements below by ticking the box associated with the number you select:

**Your pet's quality of life *before* undergoing chemotherapy**										
	**1**	**2**	**3**	**4**	**5**	**6**	**7**	**8**	**9**	**10**
	**Poor**									**Excellent**
**Your pet's quality of life *during* chemotherapy**										
	**1**	**2**	**3**	**4**	**5**	**6**	**7**	**8**	**9**	**10**
	**Poor**									**Excellent**
**Your pet's quality of life *after* undergoing chemotherapy**										
	**1**	**2**	**3**	**4**	**5**	**6**	**7**	**8**	**9**	**10**
	**Poor**									**Excellent**

8. If your pet was diagnosed with terminal cancer and you elected for a course of chemotherapy treatment, how long would you expect your pet to live for after chemotherapy treatment? Please tick the box which applies.

1–3 months	3–6 months	6–12 months	1–2 years	3–5 years	5+ years

9. Below is a list of statements, if your pet was diagnosed with terminal cancer please (tick) which answer most closely describes how you feel about the statement:

Tick the box which most closely describes how you feel about the following statements	Strongly disagree	Disagree	Neither agree or disagree	Agree	Strongly agree
Knowing the potential benefits of chemotherapy, I would opt for chemotherapy treatment for my pet					
Knowing the potential side effects of chemotherapy, I would opt for chemotherapy treatment for my pet					
I would opt for chemotherapy treatment if my pet will live for an extra 3 months with chemotherapy					
I would opt for chemotherapy treatment if my pet will live for an extra 12 months with chemotherapy					

Thank you for completing the survey.

© 2017 by the authors; licensee MDPI, Basel, Switzerland. This article is an open access article distributed under the terms and conditions of the Creative Commons by Attribution (CC-BY) license (http://creativecommons.org/licenses/by/4.0/).
